# Brain Rest

**Published:** 1886-01

**Authors:** 


**Brain Rest.** By J. LEONARD CORNING, M. D., formerly Assistant Physician to the Hudson River State Hospital for the Insane, member of the Medical Society of the county of New York, of the New York Neurological Society, etc. Second edition. Revised and enlarged with additional illustrations. New York and London: G. Putnam's Sons. 1885.

This little work is interesting, as well as instructive. The author, in his introduction, deprecates the sending abroad of all sufferers from brain exhaustion, insomnia, etc., which we heartily endorse. The sixth chapter on rest—muscular rest, spinal rest, brain rest—is exceedingly good, and, in fact, the whole work is excellent, and one which every physician will find of great value.

